# TOPK promotes the growth of esophageal cancer in vitro and in vivo by enhancing YB1/eEF1A1 signal pathway

**DOI:** 10.1038/s41419-023-05883-0

**Published:** 2023-06-16

**Authors:** Wenjie Wu, Jialuo Xu, Dan Gao, Zhenliang Xie, Wenjing Chen, Wenjing Li, Qiang Yuan, Lina Duan, Yuhan Zhang, Xiaoxiao Yang, Yingying Chen, Ziming Dong, Kangdong Liu, Yanan Jiang

**Affiliations:** 1 Pathophysiology Department, School of Basic Medical Sciences, Zhengzhou University, Zhengzhou, 450001 China; 2 The China–US (Henan) Hormel Cancer Institute, Zhengzhou, Henan 450000 China; 3 Provincial Cooperative Innovation Center for Cancer Chemoprevention, Zhengzhou University, Zhengzhou, Henan 450001 China; 4 Research Center of Basic Medical Science, Zhengzhou University, Zhengzhou, Henan 450001 China; 5 State Key Laboratory of Esophageal Cancer Prevention and Treatment, Zhengzhou, Henan 450052 China; 6 Cancer Chemoprevention International Collaboration Laboratory, Zhengzhou, Henan 450000 China

**Keywords:** Oncogenes, Immunochemistry

## Abstract

T-LAK-originated protein kinase (TOPK), a dual specificity serine/threonine kinase, is up-regulated and related to poor prognosis in many types of cancers. Y-box binding protein 1 (YB1) is a DNA/RNA binding protein and serves important roles in multiple cellular processes. Here, we reported that TOPK and YB1 were both highly expressed in esophageal cancer (EC) and correlated with poor prognosis. TOPK knockout effectively suppressed EC cell proliferation and these effects were reversible by rescuing YB1 expression. Notably, TOPK phosphorylated YB1 at Thr 89 (T89) and Ser 209 (S209) amino acid residues, then the phosphorylated YB1 bound with the promoter of the eukaryotic translation elongation factor 1 alpha 1 (eEF1A1) to activate its transcription. Consequently, the AKT/mTOR signal pathway was activated by up-regulated eEF1A1 protein. Importantly, TOPK inhibitor HI-TOPK-032 suppressed the EC cell proliferation and tumor growth by TOPK/YB1/eEF1A1 signal pathway in vitro and in vivo. Taken together, our study reveals that TOPK and YB1 are essential for the growth of EC, and TOPK inhibitors may be applied to retard cell proliferation in EC. This study highlights the promising therapeutic potential of TOPK as a target for treatment of EC.

## Introduction

The main pathological types of esophageal cancer (EC) are esophageal squamous cell carcinoma (ESCC) and esophageal adenocarcinoma (EAC). The prognosis for EC patients is poor with a 5-year survival rate of approximately 20% due to its aggressive metastatic feature and the lack of effective targeted therapies [1]. In the last decade, receptor tyrosine kinases (RTKs), including epidermal growth factor receptors (EGFRs) and vascular endothelial growth factor receptors (VEGFRs), are frequently used for most of the targeted therapies in several cancers [2]. However, these targeted treatments are virtually a failure in the therapeutic landscape for clinical EC patients. Therefore, it is urgent to exploit the molecular alterations and implement effective targeted treatments in esophageal cancer.

Proteomic and phosphoproteomic analyses provide new insights into the molecular mechanisms of cancer growth and highlight the significant targets in the therapeutic landscape for cancer patients [3–5]. The key molecules of signal pathways involved in post-translational regulation may become new potential targets for EC therapeutics in the future. T-LAK-originated protein kinase (TOPK) is a dual specificity serine/threonine and MAPKK-like kinase. Dysregulation of TOPK plays a crucial role in the proliferation, progression, and metastasis of many cancers, such as lung cancer, prostate cancer and even leukemia [6, 7]. What’s more, accumulating evidence shows that TOPK is a potential effective target for cancer-specific therapeutics by promoting cell death signaling pathways, preventing invasion, metastasis, and overcoming drug resistance [6].

Y-box binding protein 1 (YB1), a multi-functional oncoprotein with conserved Y-box protein family, is a DNA/RNA binding protein and plays an essential role in the progression of tumorigenesis [8]. YB1 promotes cell proliferation, metastasis, even drug resistance by dysregulating multiple cellular processes, such as DNA replication, transcription and repair, pre-mRNA splicing and cap-dependent mRNA translation in different cancers [8]. As a DNA-binding protein, YB1 controls the transcription of many genes involved in cell growth, including CCNB1 and EGFR [8]. More interestingly, YB1 is considered as a potent target for cancer therapy by down-regulating mRNA levels, protein expression and intervening YB1 function via various compounds [9]. Therefore, it spotlights the important significance of exploring the molecular mechanism of YB1 in tumors. Importantly, high expression of YB1 is reported to be associated with poor survival in ESCC patients, plays an oncogenic role in ESCC progression, and even is a potential target for ESCC therapy [10, 11]. However, the molecular mechanism of YB1 in the progression of EC is little known.

In our previous studies, we verified that TOPK was involved in the process of ESCC metastasis and promoted the ESCC cell mobility by activating the Src/GSK3β/STAT3 and ERK signal pathways [12]. Here, we found the protein levels of TOPK and YB1 were higher in EC tissues than in adjacent tissues and were positively related to the poor prognosis of EC patients. We also demonstrated that down-regulation of TOPK significantly inhibited EC cell proliferation by phosphorylating YB1 at T89 and S209. In comparison, phosphorylated YB1 bound with the promoter of eEF1A1 to promote the proliferation by activating AKT/mTOR signal pathway. Taken together, our study revealed an essential role of the TOPK/YB1/eEF1A1 axis in EC progression, shedding light on the molecular mechanisms and providing a theoretical basis for targeting TOPK as a promising therapeutic strategy against EC.

## Results

### Overexpressed TOPK or YB1 indicates the poor prognosis in EC patients

**Fig. 1:**
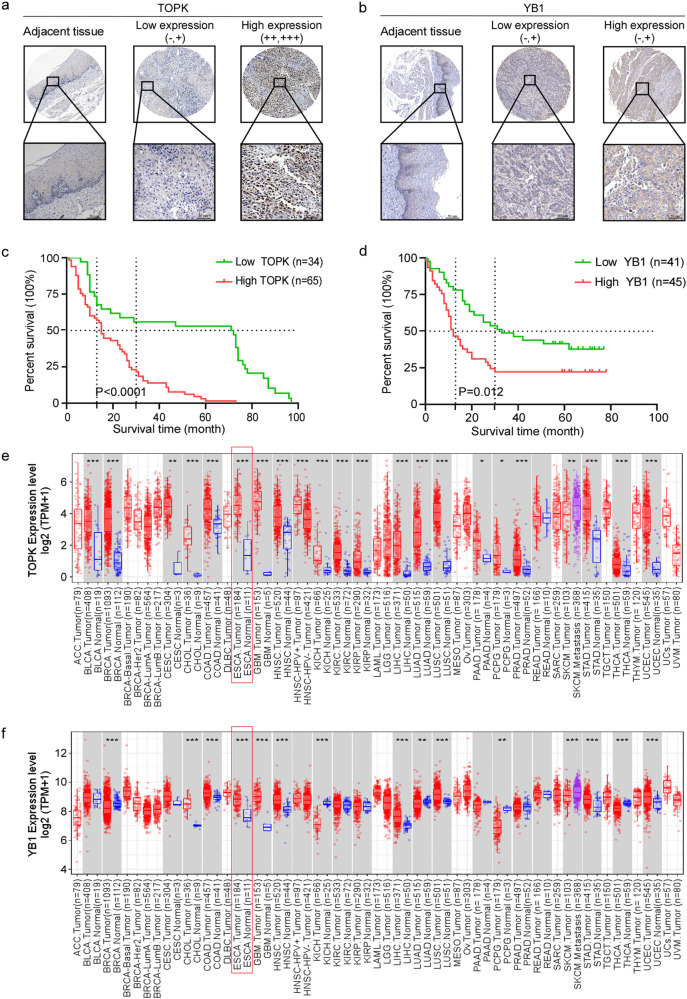
Overexpressed TOPK or YB1 indicates the poor prognosis in EC patients. **a**, **b** Representative images of IHC staining in EC tissue microarray using specific antibody for TOPK (**a**) and YB1 (**b**) in adjacent tissues and paired tumors. Scale bars represent 50 μm. **c**, **d** Survival rate of cancer patients with high or low protein levels of TOPK (**c**) and YB1 (**d**) in EC tumor microarrays. **e**, **f** Analysis of TOPK (**e**) and YB1 (**f**) mRNA levels in different cancers based on the TCGA database using the TIMER 2.0 website (TPM: Transcription per million). Red represented tumor samples, blue represented normal samples and purple represented SKCM metastasis group.

To investigate the correlation between the expression level of TOPK/YB1 protein and the clinical features of EC patients, we detected the protein levels of TOPK or YB1 in tissue microarrays. The positive expression of TOPK or YB1 in EC was primarily in the cytoplasm and/or nucleus and displayed different intensities of brown-yellow (Fig. 1a, b). These tissues were divided into a low expression group (score ≤ 3) and a high expression group (score > 3) using the criteria described previously [10, 11]. Among 100 patients, 66 patients had high expression of TOPK, 34 patients had low expression, and the adjacent tissues had negative expression. Low expression of YB1 was 50.5% (47 of 93) and high expression of YB1 was 49.5% (46 of 93) of the EC samples. There was no significant statistical correlation between high TOPK/YB1 expression and age, gender, tumor differentiation, tumor status of the EC clinical features (Tables 1 and 2). Moreover, the survival time was 19.82 ± 2.03 months in the TOPK high expression group, and 49.75 ± 5.89 months in the TOPK low expression group (Fig. 1c and Table 1). The survival time of patients with YB1 low and high expression was 38.24 ± 25.75 and 23.67 ± 24.69 months respectively (Fig. 1d and Table 2). In addition, the median survival time of TOPK low expression group was 71.5 months while the median survival time of TOPK high expression group was 15 months. The YB1 low expression group had a median survival time of 33 months while the YB1 high expression group had a median survival time of 12 months. The survival time of TOPK or YB1 expression was statistically significant by Kaplan-Meier analysis in EC (*p* < 0.001 for TOPK, *p* = 0.012 for YB1). Next, we analyzed the TOPK and YB1 mRNA levels in different tumors from the Cancer Genome Atlas (TCGA, https://www.genome.gov/Funded-Programs-Projects/Cancer-Genome-Atlas) database and found that the mRNA levels were significantly up-regulated in EC tumor tissues compared with adjacent non-cancerous tissues (Fig. 1e, f). What’s more, we found that the expression of TOPK and YB1 were both higher in several EC cell lines than in immortalized esophageal cell SHEE, and were highest in KYSE30, KYSE150 and KYSE450 (Fig. S1a). Then, we chose KYSE150 and KYSE30 cell lines, which had high TOPK expression, for the subsequent experiments. Taken together, our results indicate that TOPK and YB1 may serve as potential prognostic biomarkers for EC patients.

**Table 1:** Correlation between TOPK expression and clinicopathological characteristics of EC.

Parameters	*n*	TOPK expression		*P*-value
		Low (*n* = 34)	High (*n* = 66)	
Age, years				0.644
Mean (SD)	100	65.28 (±8.29)	65.30 (±9.76)	χ^2^ = 0.215
Gender				0.299
Male	74	23 (31.1%)	51 (68.9%)	χ^2^ = 1.081
Female	26	11 (42.3%)	15 (57.7%)	
Differentiation				0.418
I	6	3 (50.0%)	3 (50.0%)	χ^2^ = 1.745
II	67	20 (31.4%)	47 (68.6%)	
III	27	11 (37.0%)	16 (63.0%)	
Tumor status				0.644
T1-T2	11	5 (64.3%)	6 (35.7%)	χ^2^ = 0.215
T3-T4	86	27 (21.7%)	59 (78.3%)	
Lymph node status				0.023
N0	46	21 (43.48%)	25 (56.52%)	χ^2^ = 5.154
N1-N3	54	13 (25.93%)	41 (74.07%)	
Follow-up time, months				< 0.001
Mean (±SD)	100	49.75 (±5.89)	19.82 (±2.03)	χ^2^ = 27.519

**Table 2:** Correlation between YB1 expression and clinicopathological characteristics of EC.

Parameters	*n*	YB1 expression		*P*-value
		Low (*n* = 47)	High (*n* = 46)	
Age, years				0.631
Mean (SD)	93	65.32 (±9.12)	66.20 (±8.37)	
Gender				0.293
Male	77	37 (79.2%)	40 (86.7%)	χ² = 1.106
Female	16	10 (20.8%)	6 (13.3%)	
Differentiation				0.438
I	14	9 (19.1%)	5 (10.9%)	χ^2^ = 3.768
I-II	30	17 (36.2%)	13 (28.3%)	
II	30	12 (25.5%)	18 (39.1%)	
II-III	13	5 (10.6%)	8 (17.4%)	
III	5	3 (6.4%)	2 (4.3%)	
Tumor status				0.461
T1-T2	22	13 (28.9%)	9 (22.0%)	χ^2^ = 0.542
T3-T4	64	32 (71.1%)	32 (78.0%)	
Lymph node status				0.029
N0	45	28 (59.6%)	17 (37.0%)	χ^2^ = 4.762
N1-N3	48	19 (40.4%)	29 (63.0%)	
Follow-up time, months				0.009
Mean (±SD)	93	38.24 (± 25.75)	23.67 (± 24.69)	

### CRISPR/Cas9-mediated knockout of TOPK inhibits cell proliferation of EC in vitro and in vivo

**Fig. 2:**
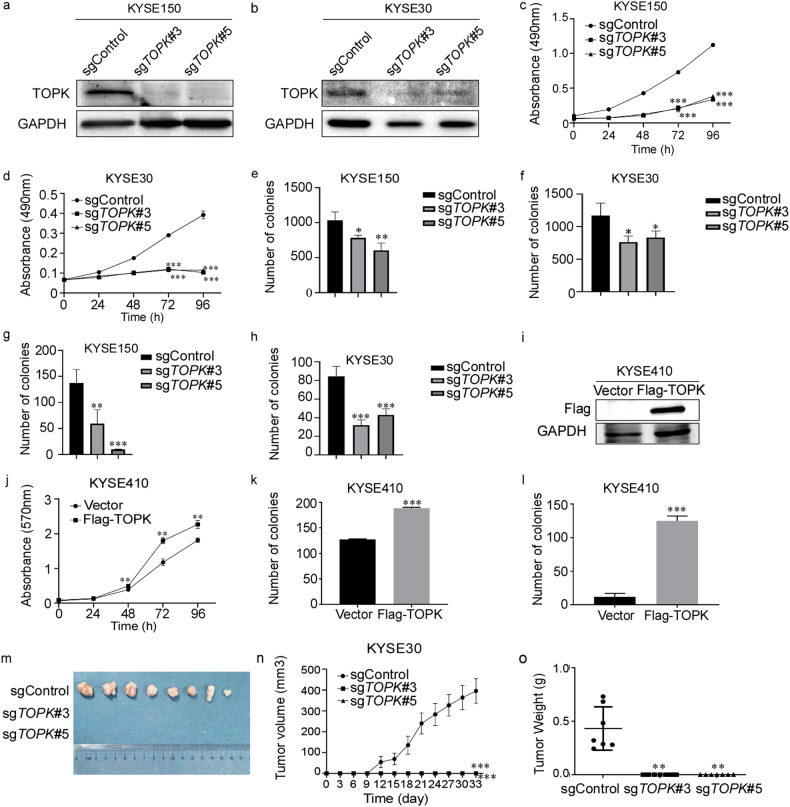
CRISPR/Cas9-mediated knockout of TOPK inhibits cell proliferation of EC in vitro and in vivo. **a**, **b** Expression levels of TOPK protein in TOPK knockout (sgRNA) KYSE150 (**a**) and KYSE30 (**b**) cells were measured by Western blot. **c**, **d** The effect of TOPK knockout on KYSE150 (**c**) and KYSE30 (**d**) cell proliferation was measured by cell proliferation assay. OD values were measured at 0, 24, 48, 72, and 96 h by MTT assay. **e**, **f** The effect of TOPK knockout on KYSE150 (**e**) and KYSE30 (**f**) cells’ anchorage-independent growth ability was measured by anchorage-independent growth assay. **g**, **h** The effect of TOPK knockout on KYSE150 (**g**) and KYSE30 (**h**) cells’ anchorage-dependent growth ability was measured by plate clone formation assay. **i** Expression levels of TOPK protein in TOPK over-expressed KYSE410 cells were measured by Western blot. **j** The effect of TOPK over-expression on KYSE410 cell proliferation was measured by cell proliferation assay. OD values were measured at 0, 24, 48, 72, and 96 h in MTT assay. **k**, **l** The effect of TOPK over-expression on KYSE410 cell’s anchorage-independent growth ability (**k**) and anchorage-dependent growth ability (**l**) in KYSE410 were measured by anchorage-independent growth assay and plate clone formation assay, respectively. **m**. The representative tumor pictures of sg*TOPK* KYSE30 CDX model. **n** The tumor growth curves in sg*TOPK* KYSE30 CDX model. **o** Tumor weights analysis of sg*TOPK* KYSE30 CDX model. All experiments were biological replicates and were repeated at least three times. Error bars showed standard error of the mean. *p ˂ 0.05, **p ˂ 0.01, ***p ˂ 0.001.

Increasingly, studies indicate that TOPK is a potential therapeutic target for tumor treatments, including osteosarcoma, lung cancer, and ovarian cancer [13]. For TOPK KO studies in EC, *TOPK* was knocked out in KYSE150 and KYSE30 cells by transfecting with two distinct CRISPR/Cas9 sgRNA lentiviral constructs (Fig. 2a, b). The proliferation was inhibited in sg*TOPK* cells compared with sgControl cells. In KYSE150, the proliferation of sg*TOPK*#3 and sg*TOPK*#5 was inhibited by 70.48% and 66.36% (Fig. 2c), and the proliferation of KYSE30 decreased by 79.04% and 76.20%, respectively (Fig. 2d). The anchorage-independent (Fig. 2e, f, S2a, b) and anchorage-dependent (Fig. 2g, h, S2c, d) colony formation ability of KYSE150 and KYSE30 cells decreased after knocking out *TOPK*. We also generated TOPK over-expressed cell lines by transferring the pLVX-IRES-3xflag TOPK full length plasmid into KYSE410 cells (Fig. 2i). As expected, high expression of TOPK accelerated cell proliferation (Fig. 2j) and promoted colony formation (Fig. 2k, l, S2e, f) in KYSE410 cells. These results showed that knocking out *TOPK* significantly inhibited cell proliferation in vitro. We further evaluated the function of TOPK by cell-line derived xenograft (CDX) model. The images of xenograft from sgControl and sgTOPK groups were displayed (Fig. 2m). Surprisingly, there were no tumors in the sgTOPK groups. This indicated that the tumorigenic capacity of EC cells was inhibited after knocking out TOPK in vivo. We also observed that the growth curves (Fig. 2n) and tumor volumes (Fig. 2o) decreased dramatically in sg*TOPK* groups compared with sgControl group. These data indicates that TOPK is an oncogene and targeting TOPK can effectively inhibit the EC growth in vitro and in vivo.

### TOPK interacts with YB1 in EC cells

**Fig. 3:**
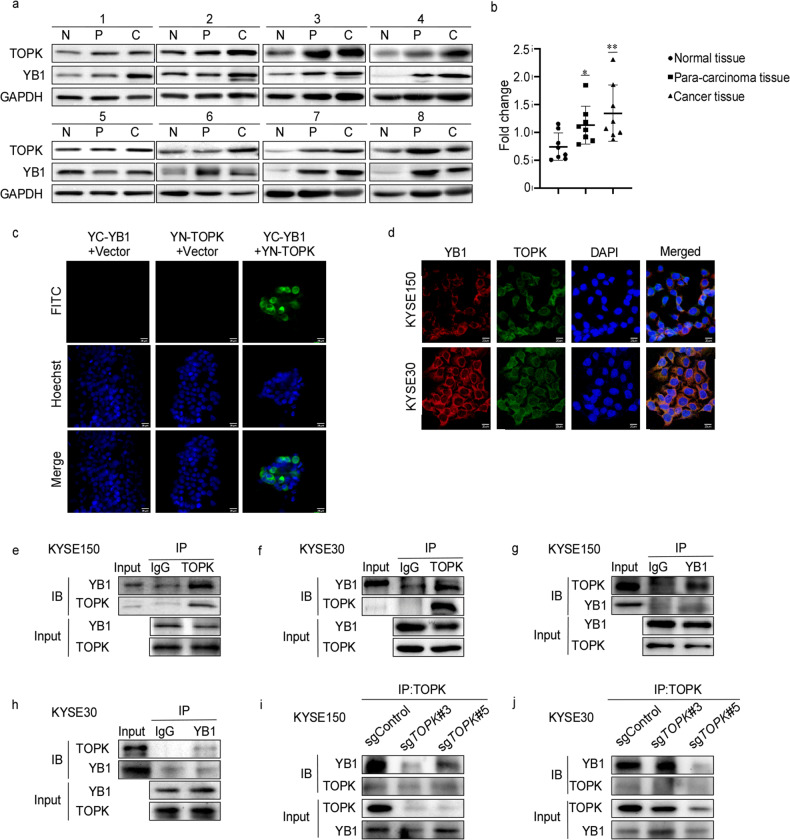
TOPK interacts with YB1 in EC cells. **a**, **b** The correlation between TOPK and YB1 protein in EC were detected by Western blot (**a**) and analyzed by image J (**b**). **c** Bimolecular fluorescence complementation (BiFC) assay was used to detect the binding between TOPK and YB1 in vitro. **d** Immunofluorescence was used to detect the binding between TOPK and YB1 in KYSE150 and KYSE30. **e**, **f** KYSE150 (**e**) and KYSE30 (**f**) cells were immunoprecipitated with anti-TOPK antibodies. After immunoprecipitation (IP) with anti-TOPK or IgG antibodies, proteins were separated by SDS-PAGE and detected by immunoblotting (IB) with anti-YB1 antibodies. The lysate was also shown as input. **g**, **h** KYSE150 (**g**) and KYSE30 (**h**) cells were immunoprecipitated with anti-YB1 antibodies. After IP with anti-YB1 or IgG antibodies, proteins were separated by SDS-PAGE and detected by immunoblotting with anti-TOPK antibodies. The lysate was also shown as input. **i**, **j** KYSE150 (**i**) and KYSE30 (**j**) cells with stable knockout of TOPK were immunoprecipitated with anti-TOPK. After IP with anti-TOPK or IgG antibodies, proteins were separated by SDS-PAGE and detected by immunoblotting with anti-YB1 antibodies. The lysate was also shown as input. All experiments were biological replicates and were repeated at least three times. Error bars showed standard error of the mean. *p ˂ 0.05, **p ˂ 0.01.

Next, we evaluated the expression of TOPK and YB1 in EC. The protein levels of TOPK and YB1 both gradually increased in normal tissues, para-cancerous tissues, and cancer tissues of EC patients (Fig. 3a). The mRNA expression between TOPK and YB1 was positively correlated by using the TCGA database (Fig. S3a). These results indicated TOPK and YB1 were positively correlated in EC tissues. To further investigate whether TOPK directly bound with YB1 in EC cells or not, we performed the BiFC assay and found that TOPK could directly bind with YB1 in cells (Fig. 3c). Moreover, TOPK and YB1 were co-located in the cytoplasm of KYSE150 and KYSE30 cells by immunofluorescence assay (Fig. 3d). Subsequently, we performed the CO-IP assay. We found that TOPK could bind with YB1 in KYSE150 (Fig. 3e) or KYSE30 (Fig. 3f) by immunoprecipitating TOPK, and YB1 could bind with TOPK in KYSE150 (Fig. 3g) or KYSE30 (Fig. 3h) by immunoprecipitating YB1. More interestingly, in KYSE150 sg*TOPK* cells, the level of YB1 which bound with TOPK decreased after knocking out TOPK (Fig. 3i). This was also observed in KYSE30 sg*TOPK* cells (Fig. 3j). Therefore, our results indicate that TOPK binds with YB1 in EC cells.

### TOPK promotes cell proliferation by phosphorylating YB1 at T89 and S209

**Fig. 4:**
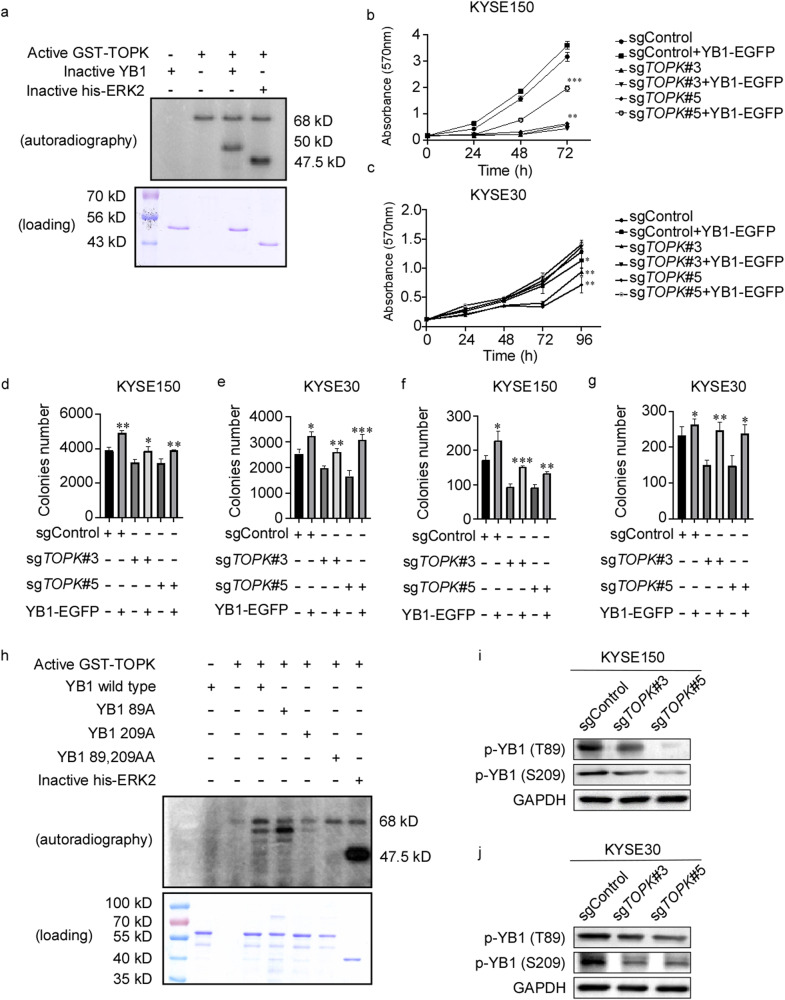
TOPK promotes cell proliferation by phosphorylating YB1 at T89 and S209. **a** YB1 kinase activity was assessed by an in vitro kinase assay using active TOPK and inactive YB1 proteins. The effect of TOPK was determined by Western blot. **b**, **c** The proliferation ability of KYSE150 (**b**) and KYSE30 (**c**) were recovered after YB1 was rescued in sg*TOPK* KYSE150 and KYSE30 cells. **d**, **e** The anchorage-independent growth ability of KYSE150 (**d**) and KYSE30 (**e**) were recovered after YB1 was rescued in sg*TOPK* KYSE150 and KYSE30 cells. **f**, **g** The anchorage-dependent growth ability of KYSE150 (**f**) and KYSE30 (**g**) were recovered after YB1 was rescued in sg*TOPK* KYSE150 and KYSE30 cells. **h** The phosphorylation ability of TOPK on YB1 was analyzed by in vitro kinase assay after mutation of T89 and S209 sites in YB1. **i**, **j** The protein level of YB1 T89A and S209A were detected in KYSE150 sg*TOPK* (**i**) and KYSE30 sg*TOPK* (**j**) cells by Western blot. All experiments were biological replicates and were repeated at least three times. Error bars showed standard error of the mean. *p ˂ 0.05, **p ˂ 0.01, ***p ˂ 0.001.

TOPK is a dual specificity serine/threonine kinase [6]. YB1 plays a primary role in tumorigenesis as a conserved transcription factor [14]. We speculated that TOPK promoted the EC cell proliferation by phosphorylating YB1. We found that TOPK could directly phosphorylate YB1 by in vitro kinase assay (Fig. 4a). These data indicated that YB1 maybe the substrate of TOPK. Next, we rescued the YB1 expression in KYSE150 sg*TOPK* and KYSE30 sg*TOPK* cells (Fig. S4a, b). After rescuing the YB1 expression, cell proliferation was increased in the rescued sg*TOPK* KYSE150 and KYSE30 cells compared with sg*TOPK* cells (Fig. 4b, c), and the anchorage-independent (Fig. 4d, e, S4c, d) and anchorage-dependent (Fig. 4f, g, S4e, f) growth ability were also enhanced by anchorage-independent growth assay and clone formation assay. These findings indicate overexpression of YB1 alleviates the inhibitory effects of TOPK in EC cells.

To determine the exact phosphorylated sites of YB1 by TOPK, we combined the in vitro kinase assay and MS analysis. It was found that the T89 and S209 of YB1 were phosphorylated by TOPK (Fig. S4g). Next, we purified the YB1 mutation protein with T89A, S209A, and double mutations 89, 209AA respectively. The phosphorylation of T89A and S209A YB1 protein by TOPK was weakened compared with WT YB1 by in vitro kinase assay (Fig. 4h). T89 of YB1 was in the cold shock domain (CSD) (aa 51-129) of YB1 which bound with DNA/RNA [14]. Consistent with these results, the levels of YB1 T89A and S209A were decreased in KYSE150 sg*TOPK* and KYSE30 sg*TOPK* cells by Western blot (Fig. 4i, j). These results show that TOPK promotes cell proliferation of EC cells by phosphorylating YB1 at the T89 and S209.

### Down-regulated TOPK inhibits YB1 binding to the promoter of eEF1A1

**Fig. 5:**
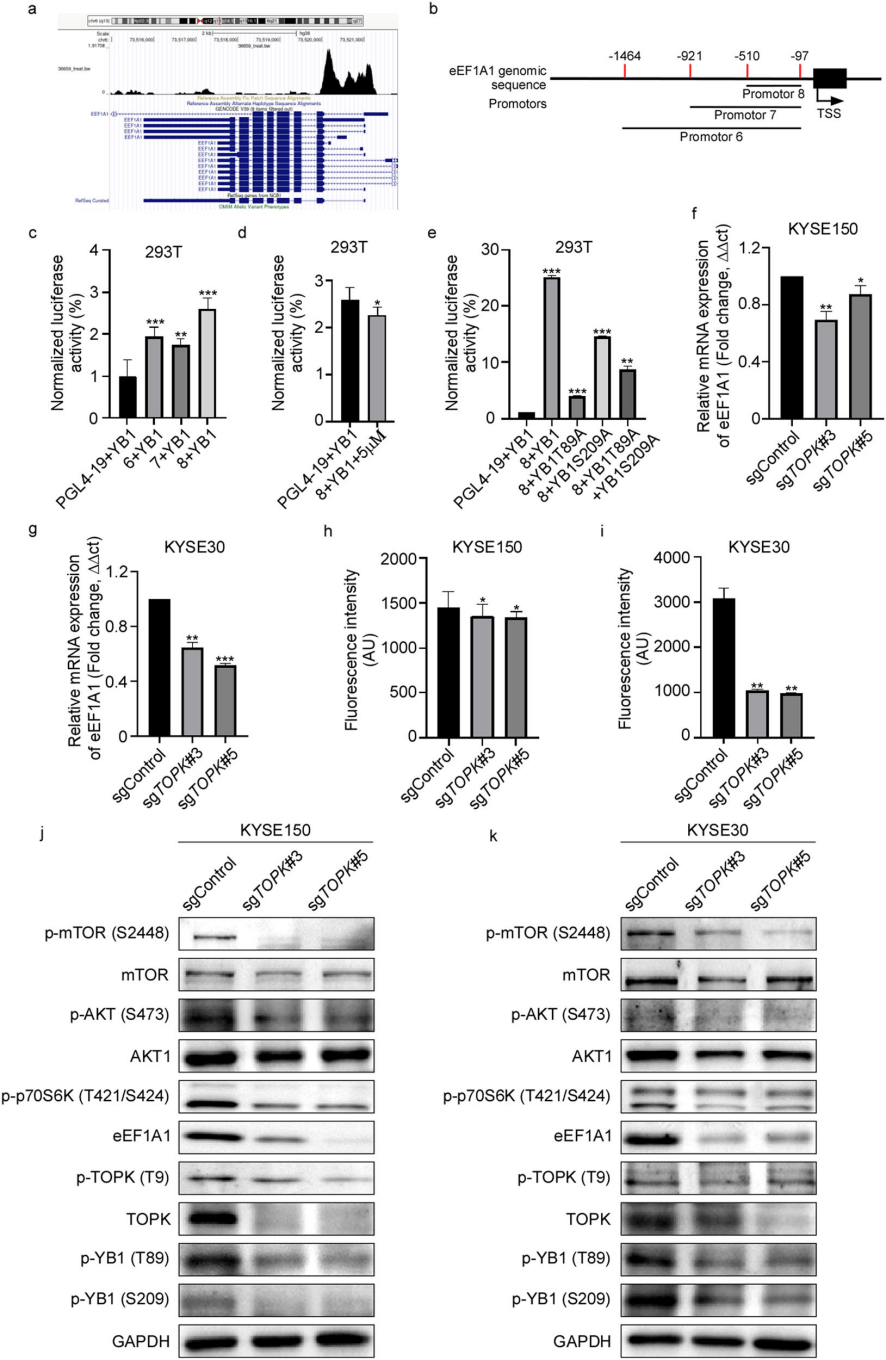
TOPK inhibits YB1 binding to the promoter of eEF1A1. **a** EEF1A1 was the top putative target of YB1 by CHIP-seq in HEK293 cell in GEO data (GSM1151769). **b** The schematic diagram of different binding sequences in the promoter of eEF1A1 designed for the dual-luciferase reporter assay. **c** The binding sequence between YB1 and eEF1A1 promoter was identified by luciferase activity assay. **d** The binding ability between YB1 and eEF1A1 promoter sequence was inhibited by TOPK inhibitor HI-TOPK-032. **e** The binding ability between YB1 and eEF1A1 promoter sequence was suppressed after YB1 mutation. **f**, **g** The mRNA level of eEF1A1 was identified in TOPK knockout KYSE150 (**f**) and KYSE30 (**g**) cells by q-PCR. **h**, **i** The general translational level of protein synthesis was confirmed in the sg*TOPK* KYSE150 (**h**) and KYSE30 (**i**) cells by protein synthesis assay. **j**, **k** The protein level of eEF1A1 and the phosphorylation levels of mTOR, AKT and p70S6K were identified in the sg*TOPK* KYSE150 (**j**) and KYSE30 (**k**) cells by Western blot. All experiments were biological replicates and were repeated at least three times. Error bars showed standard error of the mean. *p ˂ 0.05, **p ˂ 0.01, ***p ˂ 0.001.

YB1 is reported to be associated with many aspects of gene expression that cause tumor cell growth and drug resistance [15]. The cold shock domain of YB1 specifically binds to pyrimidine-rich sequences of nucleic acids (T or C) [14]. YB1 can bind to the Y/CCAAT box which is over-represented in the promoters of genes overexpressed in several cancers, including breast cancer, colon cancer, prostate cancer and leukemias [16]. In this study, we screened the target genes of YB1 by Cistrome Data Browser (http://cistrome.org). In GEO data (GSM1151769), eEF1A1 was the top putative target of YB1 by CHIP-seq in HEK293 cells (Fig. 5a). Therefore, we hypothesized that YB1 bound with the promoter of eEF1A1 in EC cells. To verify this hypothesis, we first scanned the binding motif of YB1 in the promoter of eEF1A1 (the 2 K bp sequence upstream from the transcriptional start site (TSS)) by JASPAR (https://jaspar.genereg.net/). A total of 32 putative site(s) were predicted with relative profile score threshold 80% (Table 3). According to this result, we designed different binding sequences in the promoter of eEF1A1 to do the dual-luciferase reporter assay (Fig. 5b). As expected, we found that YB1 bound with different sequences of promoter to transcript *eEF1A1* gene. And the 1–500 sequence upstream from TSS was the binding site of eEF1A1 (Fig. 5c). Next, we attested whether TOPK inhibitor HI-TOPK-032 could inhibit this transcription. We found that HI-TOPK-032 successfully inhibited the binding of YB1 to the 1–500 bp upstream in the promoter of eEF1A1 (Fig. 5d). We further transferred the mutated YB1 and found that the transcriptional efficiency was lower in the YB1 mutation groups compared with YB1 WT group (Fig. 5e). We also confirmed that the mRNA levels of eEF1A1 were decreased after knocking out TOPK in EC cells (Fig. 5f, g). Furthermore, we observed the general translational level of protein synthesis was decreased in the sg*TOPK* cells compared with the sgControl cells by protein synthesis assay (Fig. 5h, i, S5a, b). These data suggest that knocking out TOPK inhibits YB1 binding to the promoter of eEF1A1 in EC.

**Table 3:** A total of 32 putative site(s) were predicted with relative profile score threshold 80%.

Matrix ID	Name	Score	Relative score	Sequence ID	Start	End	Strand	Predicted sequence
UN0139.1	UN0139.1.YBX1	9.3550625	0.927383069	seq1	1491	1499	-	TGTACCTTC
UN0139.1	UN0139.1.YBX1	7.8363214	0.901966679	seq1	153	161	-	TGATCCACC
UN0139.1	UN0139.1.YBX1	7.2945886	0.892900689	seq1	1559	1567	+	CGTCCCTTC
UN0139.1	UN0139.1.YBX1	7.1501417	0.890483347	seq1	543	551	-	AGTTCCTTC
UN0139.1	UN0139.1.YBX1	7.0800276	0.889309975	seq1	1750	1758	-	TTCTCCACC
UN0139.1	UN0139.1.YBX1	6.0366173	0.871848327	seq1	802	810	-	CATCCTATC
UN0139.1	UN0139.1.YBX1	6.0242734	0.87164175	seq1	1865	1873	-	CGCGCCACC
UN0139.1	UN0139.1.YBX1	5.755254	0.867139662	seq1	1454	1462	-	CTTGCCATC
UN0139.1	UN0139.1.YBX1	5.1445255	0.856919021	seq1	199	207	-	GGTTTCACC
UN0139.1	UN0139.1.YBX1	4.831588	0.851681955	seq1	1582	1590	+	GTCACCACC
UN0139.1	UN0139.1.YBX1	4.7039003	0.849545084	seq1	904	912	-	TTATCCACC
UN0139.1	UN0139.1.YBX1	4.6782813	0.849116345	seq1	649	657	-	TGTTTCTTC
UN0139.1	UN0139.1.YBX1	4.4680595	0.845598248	seq1	1924	1932	-	TCCCCCACC
UN0139.1	UN0139.1.YBX1	4.431187	0.844981183	seq1	1357	1365	-	CGCCCCTCC
UN0139.1	UN0139.1.YBX1	4.012538	0.837975018	seq1	1200	1208	-	GGCTCCTTC
UN0139.1	UN0139.1.YBX1	3.5330088	0.829950017	seq1	1025	1033	+	AGTACCATG
UN0139.1	UN0139.1.YBX1	3.3405585	0.826729328	seq1	424	432	-	AGTCCTTTC
UN0139.1	UN0139.1.YBX1	3.2362	0.824982872	seq1	210	218	+	CGTCTCTTC
UN0139.1	UN0139.1.YBX1	3.0611627	0.822053592	seq1	1301	1309	+	CGACCCTTC
UN0139.1	UN0139.1.YBX1	3.0496738	0.821861323	seq1	395	403	-	TTTTCTTTC
UN0139.1	UN0139.1.YBX1	3.0064347	0.821137709	seq1	1382	1390	-	GGCTCCTCC
UN0139.1	UN0139.1.YBX1	2.9814205	0.820719093	seq1	1539	1547	-	CCCTCTACC
UN0139.1	UN0139.1.YBX1	2.8777606	0.818984328	seq1	1493	1501	+	AGGTACACC
UN0139.1	UN0139.1.YBX1	2.862601	0.818730629	seq1	391	399	-	CTTTCTTTC
UN0139.1	UN0139.1.YBX1	2.7140968	0.816245385	seq1	868	876	+	TGTGACTTC
UN0139.1	UN0139.1.YBX1	2.2386243	0.808288274	seq1	251	259	+	AGTCCCAGC
UN0139.1	UN0139.1.YBX1	2.076117	0.805568686	seq1	495	503	+	CTTCCCAAC
UN0139.1	UN0139.1.YBX1	2.0109136	0.804477496	seq1	1716	1724	+	CGCCCCCTC
UN0139.1	UN0139.1.YBX1	1.9663289	0.803731363	seq1	1891	1899	-	CACGACATC
UN0139.1	UN0139.1.YBX1	1.9048911	0.802703192	seq1	1161	1169	-	CGTAACACG
UN0139.1	UN0139.1.YBX1	1.8421957	0.801653974	seq1	10	18	-	TAAAATATC
UN0139.1	UN0139.1.YBX1	1.8211558	0.801301867	seq1	279	287	-	GATTCCTCC

EEF1A1 is a core subunit of the eukaryotic elongation factor family -- eEF1 complex that regulates protein synthesis by binding and delivering the GTP and amino acid-tRNA [17]. Aside from this, eEF1A1 has been implicated in a wide variety of cellular processes, such as nuclear tRNAs exportation, signaling transduction [18], and cellular apoptosis cytoskeleton regulation [18]. Therefore, we checked the down-regulated signal pathways in sg*TOPK* KYSE150 and KYSE30 cells. Surprisingly, we found that after knocking out TOPK in EC cells, the protein level of eEF1A1 and the phosphorylation levels of mTOR, AKT and p70S6K decreased (Fig. 5j, k). These data show that TOPK may activate AKT/mTOR signal pathways after phosphorylated YB1 binds with the promoter of eEF1A1.

### HI-TOPK-032 inhibits cell proliferation by YB1/eEF1A1 signal pathways in EC

**Fig. 6:**
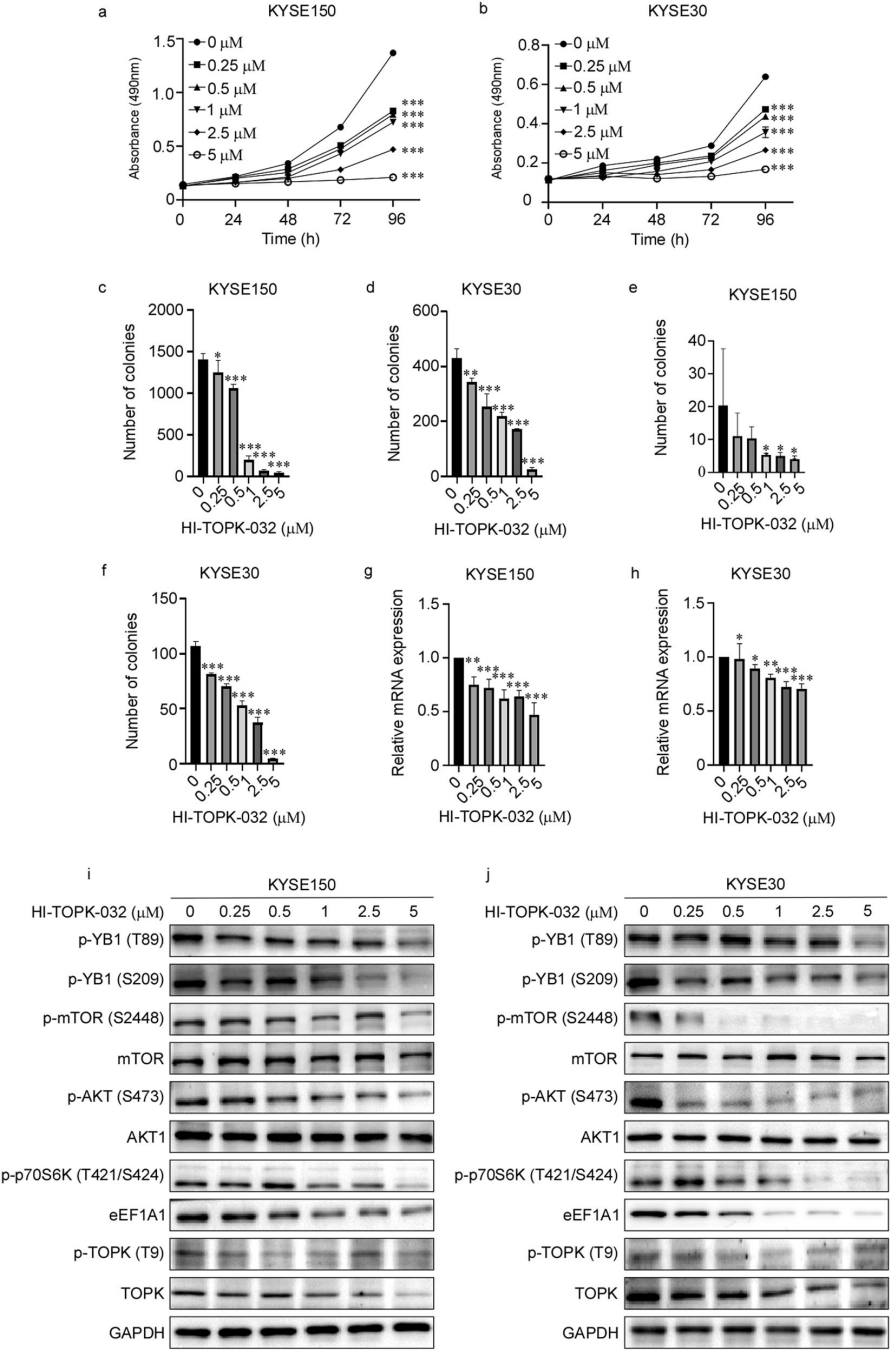
HI-TOPK-032 inhibits cell proliferation by YB1/eEF1A1 signal pathways in EC. **a**, **b** HI-TOPK-032 suppressed KYSE150 (**a**) and KYSE30 (**b**) cell viability. KYSE150 and KYSE30 cells were treated with various concentration of HI-TOPK-032 (0, 0.25, 0.5, 1, 2.5, 5 μM) and measured at 24, 48, 72 and 96 h. **c**, **d** HI-TOPK-032 suppressed anchorage-independent growth of KYSE150 (**c**) and KYSE30 (**d**) cells. KYSE150 and KYSE30 cells were treated with various concentration of HI-TOPK-032 (0, 0.25, 0.5, 1, 2.5, 5 μM) and measured after 7 days. **e**, **f** HI-TOPK-032 suppressed anchorage-dependent growth of KYSE150 (**e**) and KYSE30 (**f**) cells. KYSE150 and KYSE30 cells were treated with various concentration of HI-TOPK-032 (0, 0.25, 0.5, 1, 2.5, 5 μM) and measured after 10 days. **g**, **h**. HI-TOPK-032 decreased the mRNA level of eEF1A1 of KYSE150 (**g**) and KYSE30 (**h**) cells. KYSE150 and KYSE30 cells were treated with various concentration of HI-TOPK-032 (0, 0.25, 0.5, 1, 2.5, 5 μM). **i**, **j** The protein level of eEF1A1 and the phosphorylation levels of YB1, mTOR, AKT, and p70S6K were identified in KYSE150 (**i**) and KYSE30 (**j**) cells by Western blot. KYSE150 and KYSE30 cells were treated with various concentration of HI-TOPK-032 (0, 0.25, 0.5, 1, 2.5, 5 μM). All experiments were biological replicates and were repeated at least three times. Error bars showed standard error of the mean. *p ˂ 0.05, **p ˂ 0.01, ***p ˂ 0.001.

Currently, the previous findings support that TOPK is a therapeutical target of cancers with minimal side effects [6]. Then we evaluated whether TOPK was a potential therapeutical target for EC treatment by the TOPK inhibitor HI-TOPK-032. HI-TOPK-032 is firstly defined as a specific TOPK inhibitor for suppressing the colon cancer growth in our previous report [19]. After KYSE150 and KYSE30 cells were treated with HI-TOPK-032 (0, 0.1, 1, 10, 50, and 100 μM) for 24 h and 48 h, the viability of KYSE150 and KYSE30 was dramatically inhibited, and the IC50 of KYSE150 and KYSE30 were 13.87 and 8.34 μM at 48 h respectively (Fig. S6a, b). And we found that cell proliferation of KYSE150 (Fig. 6a) and KYSE30 (Fig. 6b) decreased gradually with the increasing concentration of HI-TOPK-032. Furthermore, the anchorage-independent growth (Fig. 6c, d, S6c, d) and anchorage-dependent growth (Fig. 6e, f, S6e, f) of KYSE150 and KYSE30 cells were suppressed in a dose-dependent manner after HI-TOPK-032 treatment. The mRNA levels of eEF1A1 were also decreased in a dose dependent manner after HI-TOPK-032 treatment (Fig. 6g, h). To verify the above molecular mechanism that TOPK promoted EC cell proliferation by phosphorylating YB1, we manifested the related signal pathways by Western blot. As expected, the protein levels of p-YB1 (T89), p-YB1 (S209), p-TOPK (T9), eEF1A1, p-AKT (S473), p-mTOR (S2448) and p-p70S6K (T421/S424) (Fig. 6i, j) decreased after HI-TOPK-032 treatment for 24 h.

### HI-TOPK-032 inhibits EC growth in vivo

**Fig. 7:**
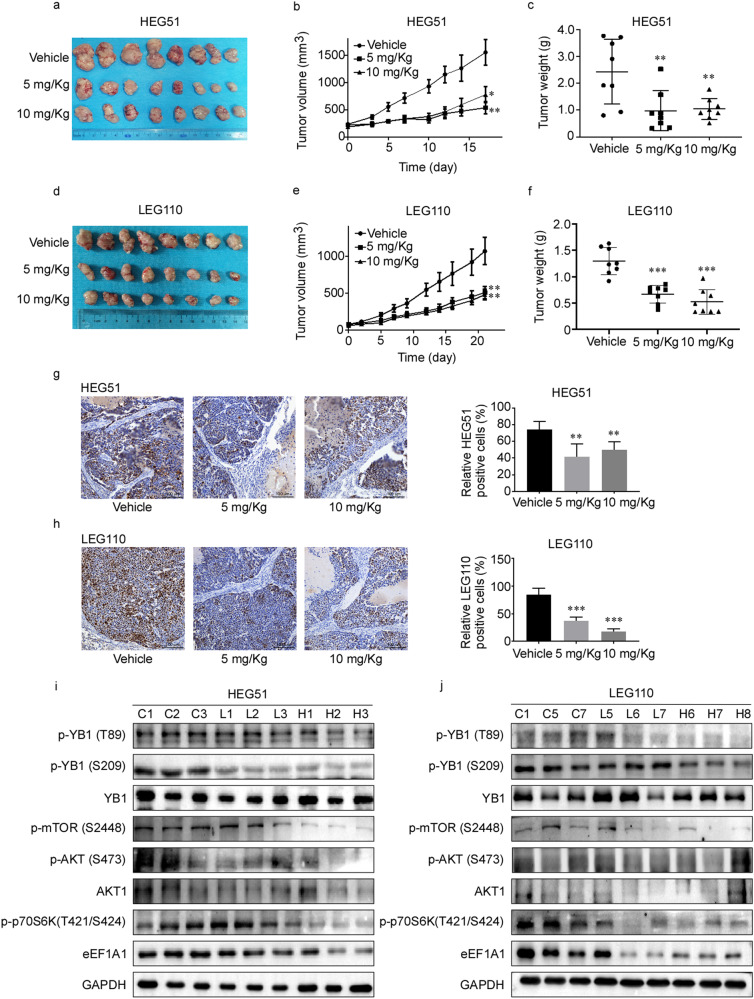
HI-TOPK-032 inhibits EC growth in vivo. **a**, **d** Representative tumor images of HEG51 (**a**) and LEG110 (**d**) after HI-TOPK-032 treatment. **b**, **e** Tumor volumes of HEG51 (**b**) and LEG110 (**e**) were recorded every two days. Tumor growth curve of each group was shown. **c**, **f** Analysis of tumor weights in HEG51 (**c**) and LEG110 (**f**) after HI-TOPK-032 treatment. **g**, **h** Representative tumor images and statistical analysis of IHC positive staining of Ki67 in HEG51 (**g**) and LEG110 (**h**) tumor tissue slices. **i**, **j** The phosphorylation levels of YB1, mTOR, AKT and p70S6K and the protein level of eEF1A1 were identified in HEG51 (**i**) and LEG110 (**j**) after HI-TOPK-032 treatment by Western blot. ‘C’ indicated vehicle group, ‘L’ indicated low treatment (5 mg/kg) group and ‘H’ indicated high treatment (10 mg/kg) group. All experiments were biological replicates and were repeated at least three times. Error bars showed standard error of the mean. *p ˂ 0.05, **p ˂ 0.01, ***p ˂ 0.001.

To further demonstrate the function of HI-TOPK-032 in suppressing the growth of EC tumor in vivo, we selected the HEG51 and LEG110 PDX models and treated them with HI-TOPK-032 (Fig. 7a, d). The tumor volumes (Fig. 7b, e) and weights were apparently decreased after HI-TOPK-032 treatment (Fig. 7c, f). The weights of mice were not changed. The expression levels of Ki67 decreased in HEG51 (Fig. 7g) and LEG110 (Fig. 7h) after HI-TOPK-032 treatment. These data indicated that HI-TOPK-032 had the sensitive inhibition to EC cancer. To identify the above molecular mechanism in EC tumors, we confirmed the related signal pathways by Western blot. After HI-TOPK-032 treatment, the protein levels of p-YB1 (T89), p-YB1 (S209), p-TOPK (T9), eEF1A1, p-AKT (S473), p-mTOR (S2448) and p-p70S6K (T421/S424) (Fig. 7i, j) in the TOPK-treated group decreased as predicted.

## Discussion

Accumulating reports have demonstrated that abnormal changes in signaling pathways are closely related to the occurrence and development of EC [20–23]. The whole genome sequencing studies show that Wnt signal pathway, Notch pathway and cell cycle-related pathways play an important role in the occurrence and development of esophageal squamous cell carcinoma [24, 25]. However, currently only EGFR and VEGFR based-target therapies have been tried clinically, and there is a lack of effective kinase targets in EC [2]. Therefore, there is an urgent need to screen the key kinases of abnormal signaling pathways in EC, and to evaluate their feasibility as therapeutic targets for EC treatments excluding the surgical resection, radiotherapy, and chemotherapy. The results of phosphorylomics, proteomics and other multi-omics studies in the last two years show that compared with the normal esophagus, the expressions of many kinases, such as CDK1, CDK2, CDC7, CHEK2 and CK2A1 are increased, providing kinase targets for targeted therapy in EC [4, 5]. Our previous studies have also approved that targeting PAK4 kinase or AURKA kinase can significantly inhibit the occurrence and development of ESCC [26, 27]. These studies show that targeting key kinases of signaling pathways is a potential treatment strategy to inhibit the occurrence and development of EC. Here, we found that TOPK and YB1 were both overexpressed and positively related with the poor prognosis of EC patients (Fig. 1c, d). Moreover, overexpressed TOPK promoted cell proliferation and tumor growth of EC cells in vitro and in vivo by YB1/eEF1A1 signal pathways.

Numerous reports have shown that YB1 is a key member of the mammalian CSD-containing protein family and has been implicated in a series of processes including cell proliferation, survival, metastasis, and drug resistance through binding to RNA and DNA and interacting with other proteins [28]. It regulates the transcription of genes involved in drug resistance, cell proliferation and progression, including MDR1, CCNA and PIK3C, MET, and CD44 [29]. Here, we found that TOPK phosphorylated YB1 at T89 and S209 to promote cell proliferation in EC. Additionally, we also demonstrated that YB1 could bind with the ‘CCATC’ sequence 500 bp upstream from the TSS to activate *eEF1A1* gene transcription (Fig. 5b). Further, HI-TOPK-032 and mutation of YB1 T89 and S209 could inhibit the transcript of eEF1A1 (Fig. 5c, d, e). Full length YB1 protein consists of 324 amino acids. The CSD domain of full length YB1 carries 2 consensus RNA recognition motifs (RRM), ribonucleoprotein particle 1 (RNP1) and RNP2 [30–32]. T89 is in the RNP2 and S209 is in the C-terminal extension of the CSD [31]. Our results demonstrate that T89 plays a primary role in the regulation of eEF1A1, while both T89 and S209 of YB1 do not function synergistically in transcriptional induction (Fig. 6e). These results were consistent that CBD domain of YB1 bound specifically with DNA/RNA sequence, while the C-terminal domain just bound non-specifically. Therefore, we speculate that YB1 binds with the promoter of eEF1A1 by mostly phosphorylating at T89. The precise molecular mechanisms should be explored in the future.

EEF1A1, one isoform of eEF1A, is a 50 kDa GTPase which couples the hydrolysis of GTP to GDP with the delivery of amino acyl tRNAs to the ribosome during protein translation [6, 8]. Recently, eEF1A1 is reported as a pleiotropic protein and is highly expressed in many cancers, including hepatocellular carcinoma, renal cell carcinoma and gastric cancer [17, 33, 34]. EEF1A1 may be a valuable prognostic biomarker and promising therapeutic target for many cancers. Knockdown of eEF1A1 attenuates proliferation and promotes the apoptosis by decreasing the level of p-AKT and p-ERK in RCC cells [34]. The clinical significance and the exact role of eEF1A1 in EC remain obscure. In our study, we found that the transcripts of eEF1A1 were activated by TOPK/YB1 signal pathway and accompanied the decreasing level of eEF1A1, AKT/mTOR signal pathways were inactivated in EC cells (Fig. 5f, i, j, k). These data indicated TOPK promoted cell proliferation of EC by targeting YB1/eEF1A1/AKT/mTOR signal pathways in vitro.

More interestingly, TOPK inhibitors, including HI-TOPK-032 [6], OTS964 [35] and OTS514 [13], may impart minimal damages to normal tissues. Studies have shown that HI-TOPK-032 which directly inhibits TOPK activity in vivo & in vitro suppresses the growth by inducing the apoptosis of colonic cancer cells. HI-TOPK-032 can also inhibit growth and survival of glioma initiating cells in vitro and even attenuated tumor growth in vivo [19, 36]. OTS514 and OTS964 have been evaluated in several solid tumors (e.g., ovarian cancer, myeloma, and cervical cancer), and play an anti-tumor role by inducing cell cycle arrest and apoptosis [37–39]. What’s more, OTS514 exhibits potent anti-myeloma activity in pre-clinical models [38]. Moreover, HI-TOPK-032 is found to suppress the progression of prolactinomas by inhibiting the phosphorylation level of the downstream target p38 MAPK [30, 40] and the solar ultraviolet-induced skin carcinogenesis by inhibiting the phosphorylating and activating c-Jun at S63 and S73 [40]. HI-TOPK-032 treatment also attenuates glioblastomas tumor growth in vitro and in vivo [36]. Here, we treated the EC cells and PDX models with HI-TOPK-032 (Fig. 7), and then found that HI-TOPK-032 significantly inhibited cell proliferation in vitro and in vivo by the TOPK/YB1/eEF1A1 signal pathway. Therefore, TOPK may become a potential therapeutic target of EC and TOPK inhibitors may be useful in combination with traditional clinical treatments for EC in the future.

In summary, TOPK and YB1 were both overexpressed and positively related with the poor prognosis of EC. And TOPK phosphorylated YB1 at T89 and S209 to promote the growth of EC, and then the phosphorylated YB1 induced the transcripts of *eEF1A1*, ultimately activated the AKT/mTOR pathways. This study provided insights into the molecular mechanisms of EC carcinogenesis and the theoretical basis for TOPK as a target in EC treatment.

## Materials and Methods

### Reagents and antibodies

Chemical reagents, including Tris, NaCl, and SDS, for molecular biology and buffer preparation were purchased from Solarbio (Beijing, China). Antibodies (Table 4) for Western blot analysis were from Cell Signaling Technology, Abcam, or Santa Cruz Biotechnology. RPMI-1640 medium, Dulbecco’s modified Eagle’s medium (DMEM, BI, USA), basal medium eagle (BME) and fetal bovine serum (FBS) were all purchased from BI (Bioind, Israel).

**Table 4:** List of antibodies.

Antibodies	Company	catalog
PBK	Santa Cruz Biotechnology	sc-390399
p-PBK/TOPK(Thr9)	Cell signaling technology	#4941 s
mTOR	Cell signaling technology	#2972 s
p-mTOR(Ser2448)	Cell signaling technology	#5536 s
AKT1	Cell signaling technology	#2938 s
p-AKT(Ser473)	Cell signaling technology	#4051 s
p-p70S6K(Thr421/Ser424)	Cell signaling technology	#9204 s
YB1	Abcam	ab76149
p-YB1(Thr89)	Absin Bioscience Inc	YBX1-T89W191012
p-YB1(Ser209)	Absin Bioscience Inc	YBX1-S209W191012
GAPDH	Proteintech Group	60004-1-Ig
β-tubulin	HUABIO	M1305-2
EF-1α1	Santa Cruz Biotechnology	sc-21758
GFP	Santa Cruz Biotechnology	sc-9996
ACTB	Beijing Zhong Shan Goldenbridge Biotechnology Company Ltd	TA-09

### Cell culture

Shantou human embryonic esophageal (SHEE) cell line were obtained from professor Enmin Li of Shantou University and were cultured in DMEM containing 10% FBS at 37 °C in an atmosphere of 5% CO_2_. EC cell lines (KYSE30, KYSE70, KYSE140, KYSE150, KYSE410, KYSE450, KYSE510) were obtained from the Chinese Academy of Sciences, HEK293T and HEK293F cells were purchased from American Type Culture Collection (ATCC; Manassas, VA). All cells were cytogenetically tested and authenticated before freezing. EC cells were maintained in RPMI-1640 medium with 10% FBS. HEK293T cells were maintained in DMEM medium supplemented with 10% FBS.

### Specimens and immunohistochemistry

Tissue microarrays were bought from Outdo Biotech (Shanghai, China), which had 100 cases of esophagus carcinoma with 80 cases of matched adjacent normal tissue for TOPK and 93 cases of esophagus carcinoma with 87 cases of matched adjacent normal tissue for YB1 (TOPK tissue array No: HEso-Squ180Sur-04; YB1 tissue array No: HEso-Squ180Sur-03). Clinical stage I II III IV (AJCC 7.0), TNM score and survival information were available. The tissue microarrays were used for immunohistochemical staining. The antibody of TOPK (1:50), YB1 (1:50), and Ki-67 (1:100) were applied to the slides and incubated at 4 °C overnight. The slides were washed and then incubated with secondary antibody, then detected by 3, 3’-diaminobenzidine (DAB). The grades of the positive cells were observed by microscope (20×) and analyzed by TissueFAXS software (Tissue Gnostics GmbH, Vienna, Austria, www.tissuegnostics.com) as follows: the ranges of intensity about the master marker (hematoxylin) and the immuno-histochemical staining were set by auto-detection of the software. All images were analyzed with the identical settings after adjustments. The expression levels of TOPK and YB1 protein were assessed and scored based on staining areas and intensity according to the methods [10, 12]. The staining results were scored based on these criteria: (i) calculate the percentage of positive tumor cells in tissues: zero (< 10%), 1 (11–25%), 2 (26–50%), 3 (51–75%), and 4 (76–100%); and (ii) observing the signal intensity of staining: zero (no signal), 1 (weak), 2 (moderate), 3 (marked). Immunoreactivity score was calculated by multiplying the score for the percentage of positive cells by the intensity score (range 0–12). The final score was stratified as - (0 score, absent), + (1–4 score, weak), ++ (5–8 score, moderate), +++ (9–12 score, strong), in this study, − to + was considered low expression, and ++ to +++ was considered high expression.

### Western blot

Total proteins were extracted from cells. Ice-cold radio immunoprecipitation assay buffer (RIPA: Solarbio, R0020) was used to treat the cells. Then, the mixture was centrifuged for 25 min at 14,000 rpm at 4 °C. The supernatants were collected and the concentration was quantified by BCA protein assay kit (Beyotime Biotechnology, Shanghai, China). Protein samples were boiled at 100 °C for 5 min. Then they were separated by sodium dodecyl sulfate-polyacrylamide gel electrophoresis (SDS-PAGE) and were transferred to the polyvinylidene fluoride (PVDF) membrane (Merck Millipore, Burlington, MA, USA). The membranes were blocked at room temperature for 1 h with skim milk dissolved in tris-buffered saline (5%). Correspondingly, primary antibodies were used to incubate the membranes for 16 h at 4 °C, then the membranes were incubated with secondary antibodies which were conjugated with horseradish peroxidase (HRP) for 2 h at room temperature. Finally, the membranes were visualized by enhanced chemiluminescence (ECL) kits (Thermo Scientific, USA).

### CRISPR/Cas9-mediated TOPK gene knockout

TOPK was knocked out in KYSE150 and KYSE30 cells by CRISPR/Cas9 system. An online CRISPR/Cas9 tool (CHOPCHOP: https://chopchop.cbu.uib.no/) was used to design TOPK-specific RNA guides [41]. Two oligonucleotide sequences of TOPK single guide RNA (sgRNA) were: 5’-GGAGAATGAGACAAACCTCT-3’ and 5’-TGTTTCAGTGACTGACCCT G-3’. Packaging vectors including pMD2.G, psPAX2, and viral plasmids were co-transfected with jet PRIME (Polyplus, FR) into HEK293T cells. Collection of viral particles was done at 24 h, 48 h, and 72 h post-transfection, and all viral particles were filtered by a 0.22 μm filter. Then after combination with 8 μg/mL polybrene, the viral particles were used to infect KYSE150 and KYSE30 cells. Then the infected cells were selected with puromycin (2 μg/mL) for 72 h respectively. The efficacies of sg*TOPK* in EC cells were determined by Western blot.

### Dual-luciferase reporter assay

The value of firefly-luciferase was normalized to the value of Renilla-luciferase in relative luciferase activity assay [42]. In summary, 2 × 10^4^ HEK293T cells were seeded into the wells of a 24-well plate and cultured overnight. 0.5 μg pGL4.19-eEF1A1 promoter plasmid and 0.05 μg hRluc/TK plasmid were co-transfected into cells using jet PRIME transfection reagent for 24 h, the cells were then treated with 5 μM HI-TOPK-032 for another 24 h. Dual-luciferase reporter assay system (Promega, E1910, USA) was used to detect the luciferase activity. Cells were lysed in passive lysis buffer and transferred to a 96-well plate, then firefly-luciferase and renilla-luciferase values were measured by Tecan Spark Multi-Detection System.

### Protein synthesis assay

TOPK knockout KYSE150 and KYSE30 cells (6 × 10^3^ cells/well) were seeded in 96-well plates and incubated for 16 h. Then the protein synthesis assay was performed according to the protocol of Click-iT^TM^ Plus OPP Alexa Fluor^TM^ 488 Protein Synthesis Assay Kit (C10456, Thermo Fisher Scientific). All the fluorescence intensities were detected and analyzed by In Cell Analyzer 6000.

### Cell proliferation assay

KYSE 150 and KYSE30 cells were seeded into 96-well plates with 1.5 × 10^3^ cells/well and incubated for 16 h. After incubation for another 24, 48, 72 or 96 h, methyl thiazolyl tetrazolium (MTT) reagent (0.5 mg/mL) was added to 96-well plates to detect the proliferation of EC cells. Absorbance was measured at 490 or 570 nm.

### Anchorage-independent growth assay

According to the standard method, the anchorage-independent cell growth was detected by the soft agar assay [43]. In brief, KYSE150 and KYSE30 cells (8000 cells/well) in a 6-well plate were seeded in 1 mL of 0.33% BME agar containing 10% FBS over 3 mL of 0.5% BME agar containing 10% FBS. Cells were cultured for an additional 10 days. Cells were photographed and counted by IN Cell Analyzer 6000 and Image-Pro Plus v6.0 software respectively.

### Anchorage-dependent growth assay

KYSE150 and KYSE30 cells (200 cells/well) were seeded into 6-well plates with 2 mL of medium. After 10 days’ incubation, clones were fixed by 4% paraformaldehyde and stained by 0.5% crystal violet for 5 min. Clones were photographed and counted.

### Co-immunoprecipitation (CO-IP)

KYSE150 and KYSE30 cells were collected and incubated with lysis buffer (50 mM Tris-HCl pH 7.4, 1 mM EDTA, 1% NP40, 150 mM NaCl, protease inhibitors and PMSF) for 40 min at 4 °C. KYSE150 and KYSE30 cell lysates were collected and quantified by BCA kit. Appropriate cell lysates were precleared by protein A/G agarose beads (#sc-2003, Santa Cruz) for 2 h at 4 °C. Then the cell lysates were incubated with TOPK and YB1 antibodies, then 30 μL of protein A/G agarose beads were added to each sample and rotated at 4 °C overnight. The beads were washed and boiled at 95 °C for 5 min with 6 × loading buffer. The immunoprecipitated complexes were detected by Western blot.

### Bimolecular fluorescence complementation (BiFC) assay

The full length of TOPK or YB1 was cloned to the pBiFC-VC155 or pBiFC-VN173 vector respectively. Experiments were carried out in 6 well plates (Cell carrier™ Perkin Elmer). HEK293T cells were seeded at density of 5 × 10^5^ cells per well. Cells were incubated at 37 °C with 5% of CO_2_ for 16 h. Then these plasmids were transferred to the corresponding wells. After culturing for another 48 h, cells were fixed with 4% paraformaldehyde (PFA). Then these cells were imaged by IN Cell Analyzer 6000.

### KYSE30 sg*TOPK* cell-derived xenograft (CDX) mouse models

Severe combined immunodeficient (SCID) mice (Female, 6–8 weeks old) were purchased from Vital River Labs (Beijing, China) and maintained under specific pathogen-free conditions. Mice were divided into 3 groups: 1) KYSE30 sgControl; 2) KYSE30 sg*TOPK*#3; 3) KYSE30 sg*TOPK*#5 (8 per group). After acquiring enough sgControl, sg*TOPK*#3, and sg*TOPK*#5 KYSE30 cells, 2 × 10^6^/mL cells (100 μL) were inoculated subcutaneously into the right flanks of all mice. Body weights and tumor volumes were measured 3 times every week, and tumor volumes were calculated based on the formula: tumor volume (mm^3^) = length × width × width/2. All animal experiments were performed according to guidelines approved by the Ethics Committee of Zhengzhou University (Zhengzhou, Henan, China).

### EC patient-derived xenograft (PDX) models

SCID mice (Female, six-week) were bought from Vital River Labs (Beijing, China). All mice were fed with free access to food and water and kept in 12 h/12 h light/dark cycles. EC PDX models HEG51 and LEG110 were studied in animal experiments and the information of two PDX cases was listed in Table 5. The corresponding PDX tumor tissues were separated into fragments and implanted subcutaneously into the right flanks of mice. When tumor volumes reached about 100 mm^3^, the mice were randomly divided into 3 groups:1) vehicle; 2) 5 mg/kg and 3) 10 mg/kg HI-TOPK-032 (8 per group). These mice were treated by intraperitoneal (i.p.) injection three times per week. The body weights and tumor volumes were measured every 2 days. Tumor volumes were calculated using the following formula: tumor volume (mm^3^) = length × width × width/2. Mice were monitored and euthanized when tumor volume reached about 1000 mm^3^, and then the tumors were extracted. Animal experiments in this research were approved by the Ethics Committee of Zhengzhou University (Zhengzhou, Henan, China).

**Table 5:** The information of two PDX cases.

Case	Type	Gender	Age	T	N	M	Pathological grade
HEG51	EAC	Male	53	3	2	0	IIIb
LEG110	ESCC	Male	69	3	1	0	IIIb

### Statistical analysis

In this study, SPSS (version 21.0; SPSS, Chicago, IL, USA) software was used to conduct all statistical analyses, and quantitative results were shown as mean ± SD. The unpaired Student’s t-test or one-way analysis of variance (ANOVA) was used to compare significant differences. *P*-value lower than 0.05 were considered to indicate statistical significance. The asterisk (*), (**) and (***) indicated a significance of (*p* ˂ 0.05), (*p* ˂ 0.01) and (*p* ˂ 0.001), respectively.

## Supplementary information

Supplementary figures

Reproducibility checklist

## Data Availability

The data sets used and analyzed during the current study are available from the corresponding author on reasonable request.

## References

[1] Bregni G, Beck B (2022). Toward targeted therapies in esophageal cancers: An overview. Cancers (Basel).

[2] Yang YM, Hong P, Xu WW, He QY, Li B (2020). Advances in targeted therapy for esophageal cancer. Signal Transduct Target Ther.

[3] Liu W, Xie L, He YH, Wu ZY, Liu LX, Bai XF (2021). Large-scale and high-resolution mass spectrometry-based proteomics profiling defines molecular subtypes of esophageal cancer for therapeutic targeting. Nat Commun.

[4] Li Y, Yang B, Ma Y, Peng X, Wang Z, Sheng B (2021). Phosphoproteomics reveals therapeutic targets of esophageal squamous cell carcinoma. Signal Transduct Target Ther.

[5] Jin X, Liu L, Wu J, Jin X, Yu G, Jia L (2021). A multi‐omics study delineates new molecular features and therapeutic targets for esophageal squamous cell carcinoma. Clin Transl Med.

[6] Herbert KJ, Ashton TM, Prevo R, Pirovano G, Higgins GS (2018). T-LAK cell-originated protein kinase (TOPK): an emerging target for cancer-specific therapeutics. Cell Death Dis.

[7] Huang H, Lee MH, Liu K, Dong Z, Ryoo Z, Kim MO (2021). PBK/TOPK: An effective drug target with diverse therapeutic potential. Cancers (Basel).

[8] Liu X, Chen D, Chen H, Wang W, Liu Y, Wang Y (2021). YB1 regulates miR-205/200b-ZEB1 axis by inhibiting microRNA maturation in hepatocellular carcinoma. Cancer Commun.

[9] Yang JW, Sun C, Jin QY, Qiao XH, Guo XL (2021). Potential therapeutic strategies for targeting Y-Box-binding protein 1 in cancers. Curr Cancer Drug Targets.

[10] Li Y, Wen ZS, Yang HX, Luo RZ, Zhang Y, Zhang MF (2011). High expression of Y-box-binding protein-1 is associated with poor survival in resectable esophageal squamous cell carcinoma. Ann Surg Oncol.

[11] Xu J, Hu Z (2016). Y-box-binding protein 1 promotes tumor progression and inhibits cisplatin chemo-sensitivity in esophageal squamous cell carcinoma. Biomed Pharmacother.

[12] Jiang Y, Zhang J, Zhao J, Li Z, Chen H, Qiao Y (2019). TOPK promotes metastasis of esophageal squamous cell carcinoma by activating the Src/GSK3β/STAT3 signaling pathway via γ-catenin. BMC Cancer.

[13] Thanindratarn P, Wei R, Dean DC, Singh A, Federman N, Nelson SD (2021). T-LAK cell-originated protein kinase (TOPK): an emerging prognostic biomarker and therapeutic target in osteosarcoma. Mol Oncol.

[14] Kljashtorny V, Nikonov S, Ovchinnikov L, Lyabin D, Vodovar N, Curmi P (2015). The cold shock domain of YB-1 segregates RNA from DNA by non-bonded interactions. PLoS One.

[15] Wu J, Stratford AL, Astanehe A, Dunn SE (2007). YB-1 is a transcription/translation factor that orchestrates the oncogenome by hardwiring signal transduction to gene expression. Transl Oncogenomics.

[16] Dolfini D, Mantovani R (2013). Targeting the Y/CCAAT box in cancer: YB-1 (YBX1) or NF-Y. Cell Death Differ.

[17] Chen SL, Lu SX, Liu LL, Wang CH, Yang X, Zhang Z (2018). eEF1A1 overexpression enhances tumor progression and indicates poor prognosis in hepatocellular carcinoma. Transl Oncol.

[18] Abbas W, Kumar A, Herbein G (2015). The eEF1A proteins: At the crossroads of oncogenesis, apoptosis, and viral infections. Front Oncol.

[19] Kim DJ, Li Y, Reddy K, Lee MH, Kim MO, Cho YY (2012). Novel TOPK inhibitor HI-TOPK-032 effectively suppresses colon cancer growth. Cancer Res.

[20] Kadian LK, Arora M, Prasad CP, Pramanik R, Chauhan SS (2022). Signaling pathways and their potential therapeutic utility in esophageal squamous cell carcinoma. Clin Transl Oncol.

[21] Hou S, Hao X, Li J, Weng S, Wang J, Zhao T (2022). TM4SF1 promotes esophageal squamous cell carcinoma metastasis by interacting with integrin α6. Cell Death Dis.

[22] Xing H, Gao M, Wang Y, Zhang X, Shi J, Wang X (2022). Genome-wide gain-of-function screening identifies EZH2 mediating resistance to PI3Kα inhibitors in esophageal squamous cell carcinoma. Clin Transl Med.

[23] Feng Y, Ma Z, Pan M, Xu L, Feng J, Zhang Y (2022). WNT5A promotes the metastasis of esophageal squamous cell carcinoma by activating the HDAC7/SNAIL signaling pathway. Cell Death Dis.

[24] Song Y, Li L, Ou Y, Gao Z, Li E, Li X (2014). Identification of genomic alterations in esophageal squamous cell cancer. Nature..

[25] Gao YB, Chen ZL, Li JG, Hu XD, Shi XJ, Sun ZM (2014). Genetic landscape of esophageal squamous cell carcinoma. Nat Genet.

[26] Bao Z, Li A, Lu X, Wang Z, Yu Y, Wu W (2022). Oxethazaine inhibits esophageal squamous cell carcinoma proliferation and metastasis by targeting aurora kinase A. Cell Death Dis.

[27] Wei Y, Wu W, Jiang Y, Zhou H, Yu Y, Zhao L (2022). Nuplazid suppresses esophageal squamous cell carcinoma growth in vitro and in vivo by targeting PAK4. Br J Cancer.

[28] Zhang J, Fan JS, Li S, Yang Y, Sun P, Zhu Q (2020). Structural basis of DNA binding to human YB-1 cold shock domain regulated by phosphorylation. Nucleic Acids Res.

[29] Yang XJ, Zhu H, Mu SR, Wei WJ, Yuan X, Wang M (2019). Crystal structure of a Y-box binding protein 1 (YB-1)–RNA complex reveals key features and residues interacting with RNA. J Biol Chem.

[30] Prabhu L, Hartley AV, Martin M, Warsame F, Sun E, Lu T (2015). Role of post-translational modification of the Y box binding protein 1 in human cancers. Genes Dis.

[31] Zasedateleva OA, Krylov AS, Prokopenko DV, Skabkin MA, Ovchinnikov LP, Kolchinsky A (2002). Specificity of mammalian Y-box binding protein p50 in interaction with ss and ds DNA analyzed with generic oligonucleotide microchip. J Mol Biol.

[32] Du J, Zhang G, Qiu H, Yu H, Yuan W (2020). A novel positive feedback loop of linc02042 and c-Myc mediated by YBX1 promotes tumorigenesis and metastasis in esophageal squamous cell carcinoma. Cancer Cell Int.

[33] Li X, Li J, Li F (2017). P21 activated kinase 4 binds translation elongation factor eEF1A1 to promote gastric cancer cell migration and invasion. Oncol Rep.

[34] Bao Y, Zhao TL, Zhang ZQ, Liang XL, Wang ZX, Xiong Y (2020). High eukaryotic translation elongation factor 1 alpha 1 expression promotes proliferation and predicts poor prognosis in clear cell renal cell carcinoma. Neoplasma..

[35] Yang Y, Teng QX, Wu ZX, Wang JQ, Lei ZN, Lusvarghi S (2022). PBK/TOPK inhibitor OTS964 resistance is mediated by ABCB1-dependent transport function in cancer: in vitro and in vivo study. Mol Cancer.

[36] Joel M, Mughal AA, Grieg Z, Murrell W, Palmero S, Mikkelsen B (2015). Targeting PBK/TOPK decreases growth and survival of glioma initiating cells in vitro and attenuates tumor growth in vivo. Mol Cancer.

[37] Ikeda Y, Park JH, Miyamoto T, Takamatsu N, Kato T, Iwasa A (2016). T-LAK cell-originated protein kinase (TOPK) as a prognostic factor and a potential therapeutic target in ovarian cancer. Clin Cancer Res.

[38] Stefka AT, Johnson D, Rosebeck S, Park JH, Nakamura Y, Jakubowiak AJ (2020). Potent anti-myeloma activity of the TOPK inhibitor OTS514 in pre-clinical models. Cancer Med.

[39] Ma H, Han F, Yan X, Qi G, Li Y, Li R (2021). PBK promotes aggressive phenotypes of cervical cancer through ERK/c-Myc signaling pathway. J Cell Physiol..

[40] Roh E, Han Y, Reddy K, Zykova TA, Lee MH, Yao K (2020). Suppression of the solar ultraviolet-induced skin carcinogenesis by TOPK inhibitor HI-TOPK-032. Oncogene.

[41] Wu X, Wang Z, Jiang Y, Zhou H, Li A, Wei Y (2021). Tegaserod Maleate inhibits esophageal squamous cell carcinoma proliferation by suppressing the peroxisome pathway. Front Oncol.

[42] Wu Q, Liu F, Ge M, Laster KV, Wei L, Du R (2022). BRD4 drives esophageal squamous cell carcinoma growth by promoting RCC2 expression. Oncogene..

[43] Jia X, Huang C, Hu Y, Wu Q, Liu F, Nie W (2021). Cirsiliol targets tyrosine kinase 2 to inhibit esophageal squamous cell carcinoma growth in vitro and in vivo. J Exp Clin Cancer Res.

