# Supplementary material for: TOPK promotes the growth of esophageal cancer in vitro and in vivo by enhancing YB1/eEF1A1 signal pathway
Source: Cell Death Dis. 2023 Jun 16;14(6):364. doi: 10.1038/s41419-023-05883-0 (PMC10276051; doi:10.1038/s41419-023-05883-0)
Supplement: Supplementary file 1 — Supplementary figures [file 41419_2023_5883_MOESM1_ESM.docx]

**Supplementary figures for**

**TOPK promotes the growth of esophageal cancer *in vitro* and *in vivo* by enhancing YB1/eEF1A1 signal pathway**

Wenjie Wu^# 1,2^, Jialuo Xu^# 1^, Dan Gao^1^, Zhenliang Xie^1^, Wenjing Chen^1^, Wenjing Li^1, 2^, Qiang Yuan^1, 2^, Lina Duan^1, 2^, Yuhan Zhang^1, 2^, Xiaoxiao Yang^1, 2^, Yingying Chen^1^, Ziming Dong^1,3^, Kangdong Liu* ^1,^ ^2, 3, 4, 5, 6^, Yanan Jiang* ^1, 2, 4,5^

1 Pathophysiology Department, School of Basic Medical Sciences, Zhengzhou University, Zhengzhou, China

2 The China–US (Henan) Hormel Cancer Institute, Zhengzhou, Henan, 450001, China

3 Provincial Cooperative Innovation Center for Cancer Chemoprevention, Zhengzhou University, Zhengzhou, Henan, China

4 Research Center of Basic Medical Science, Zhengzhou University, Zhengzhou, Henan, 450001, China

5 State Key Laboratory of Esophageal Cancer Prevention and Treatment, Zhengzhou, Henan, China

6 Cancer Chemoprevention International Collaboration Laboratory, Zhengzhou, Henan, China

# These authors contributed equally to this work.

*Correspondence: Yanan Jiang (yananjiang@zzu.edu.cn)

Kangdong Liu (kdliu@zzu.edu.cn)

**
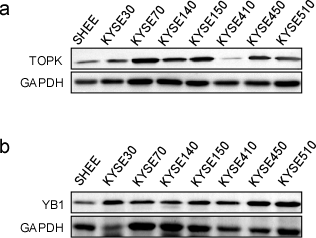
**

Supplemental Figure 1. Analysis of TOPK (a) and YB1 (b) protein levels in in SHEE and EC cell lines by Western blot. a. The expression levels of TOPK protein in SHEE and EC cell lines, including KYSE30, KYSE70, KYSE140, KYSE150, KYSE410, KYSE450 and KYSE510, were identified by Western blot. b. The expression levels of YB1 protein in SHEE and EC cell lines, including KYSE30, KYSE70, KYSE140, KYSE150, KYSE410, KYSE450 and KYSE510, were identified by Western blot. All experiments were biological replicates and were repeated at least three times.


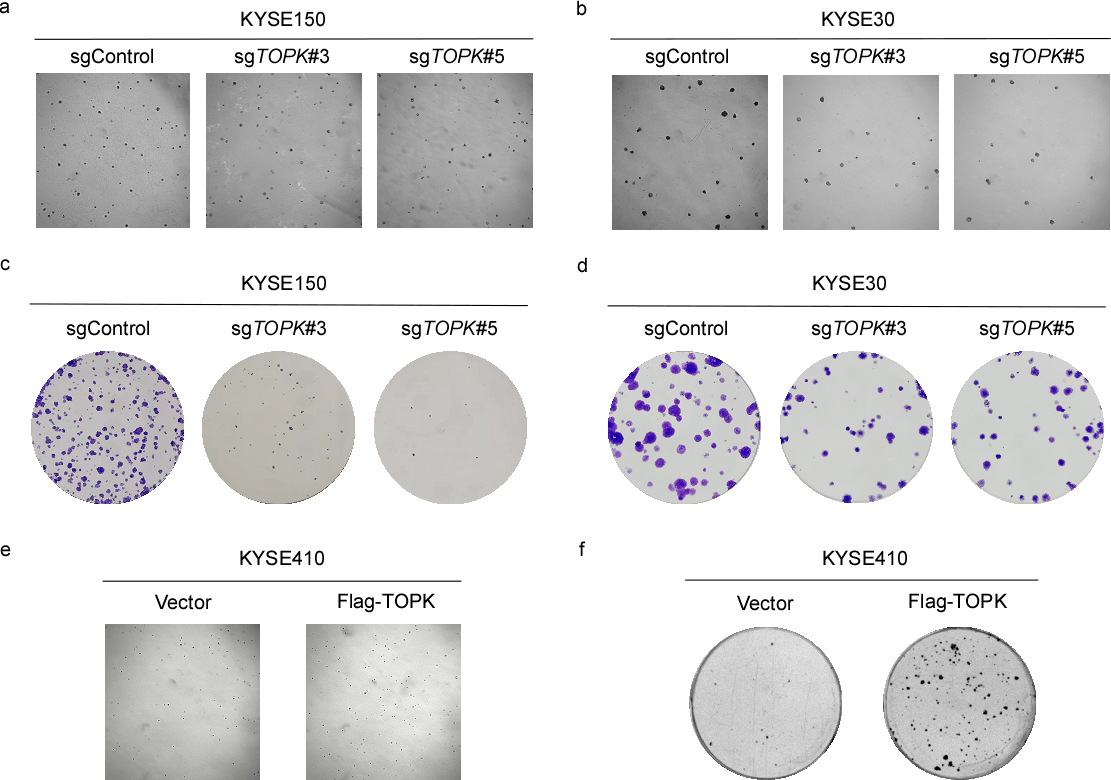


Supplemental Figure 2. Effect of CRISPR/Cas9-mediated knockout of TOPK on cell proliferation of EC *in vitro*. a b. Representative images of anchorage-independent growth in sg*TOPK* KYSE150 (a) and KYSE30 (b). c d. Representative images of anchorage-dependent growth in sg*TOPK* KYSE150 (c) and KYSE30 (d). e f. Representative images of TOPK overexpression in anchorage-independent growth (e) and anchorage-dependent growth (f) in KYSE410. All experiments were biological replicates and were repeated at least three times.


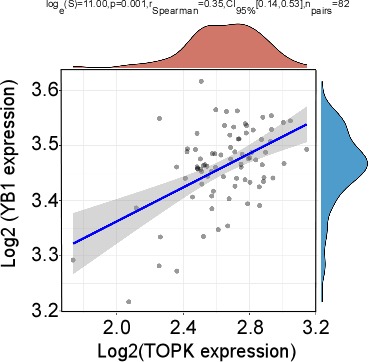


Supplemental Figure 3. The correlation of mRNA level between TOPK and YB1 in TGCA database.


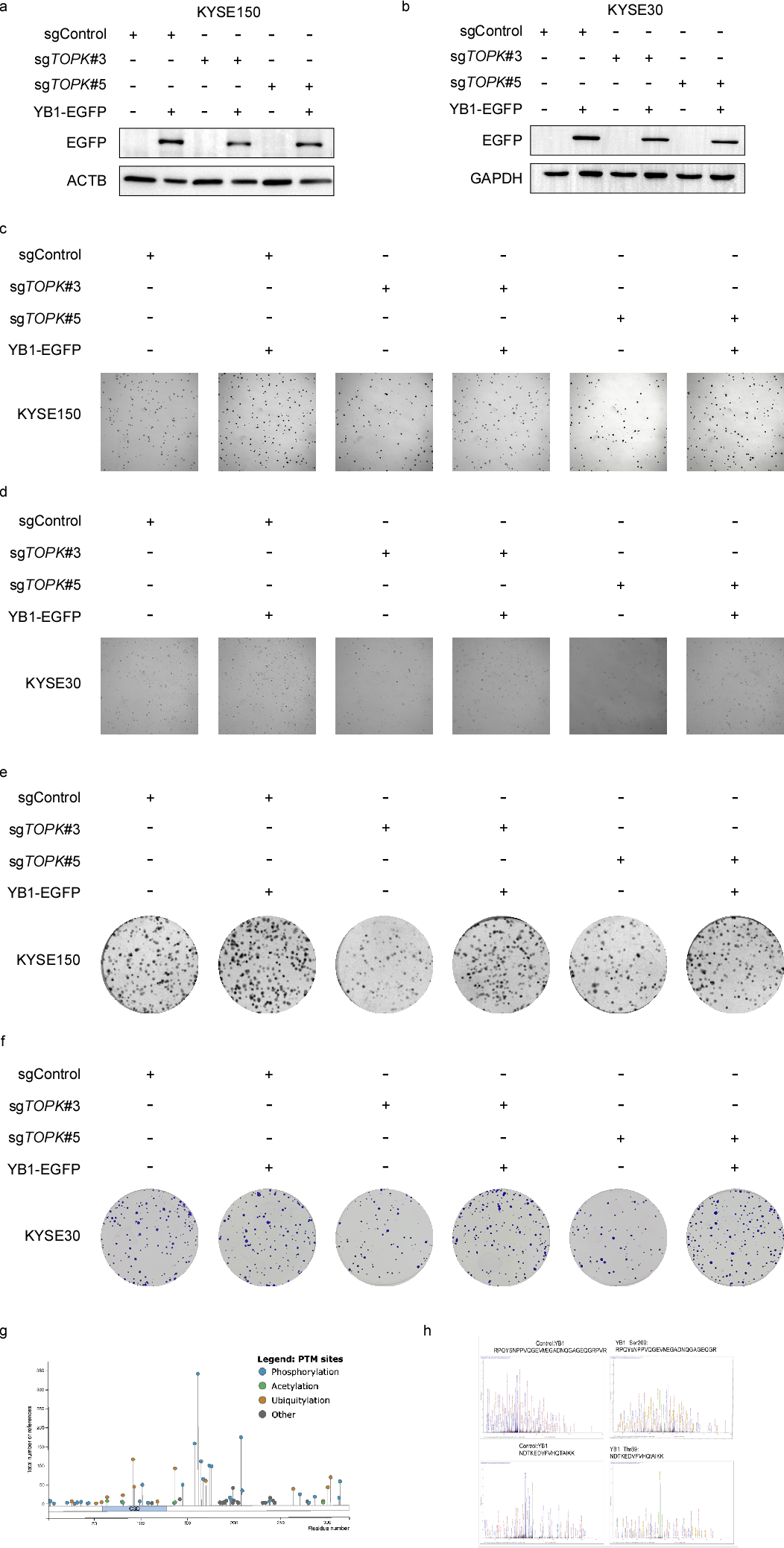


Supplemental Figure 4. The effect of rescued YB1 expression in TOPK knockout KYSE150 and KYSE30 cells on anchorage-independent growth and anchorage-dependent growth. a b. The expression level of EGFP-YB1 was rescued in sg*TOPK* KYSE150 (a) and KYSE30 (b) cells and detected by Western blot. c d. Representative images of anchorage-independent growth in YB1 recovered sg*TOPK* KYSE150 (c) and KYSE30 (d) cells. e f. Representative images of anchorage-dependent growth in YB1 recovered sg*TOPK* KYSE150 (e) and KYSE30 (f) cells. g. The exact phosphorylated sites of YB1. h. The phosphorylation of YB1 T89A and S209A protein by TOPK kinase decreased compared with WT YB1 by *in vitro* kinase assay. All experiments were biological replicates and were repeated at least three times.


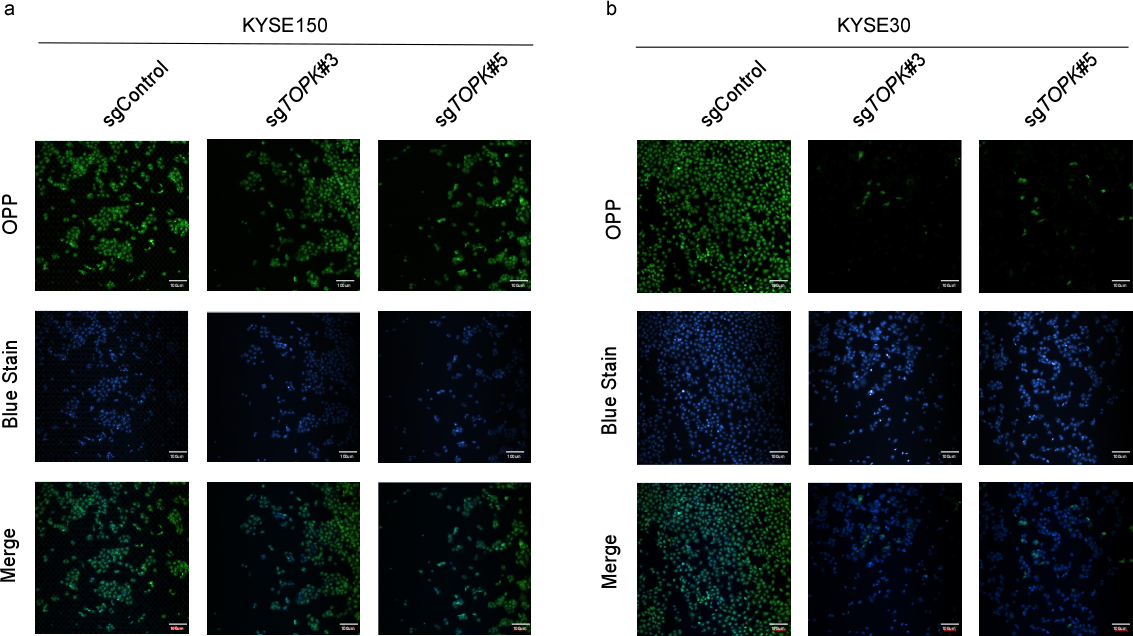


Supplemental Figure 5. The general translational level of protein synthesis was identified in the sg*TOPK* KYSE150 (a) and KYSE30 (b) cells by protein synthesis assay. a b. Representative images of general translational level of protein synthesis in sg*TOPK* KYSE150 (a) and KYSE30 (b) cells. All experiments were biological replicates and were repeated at least three times.


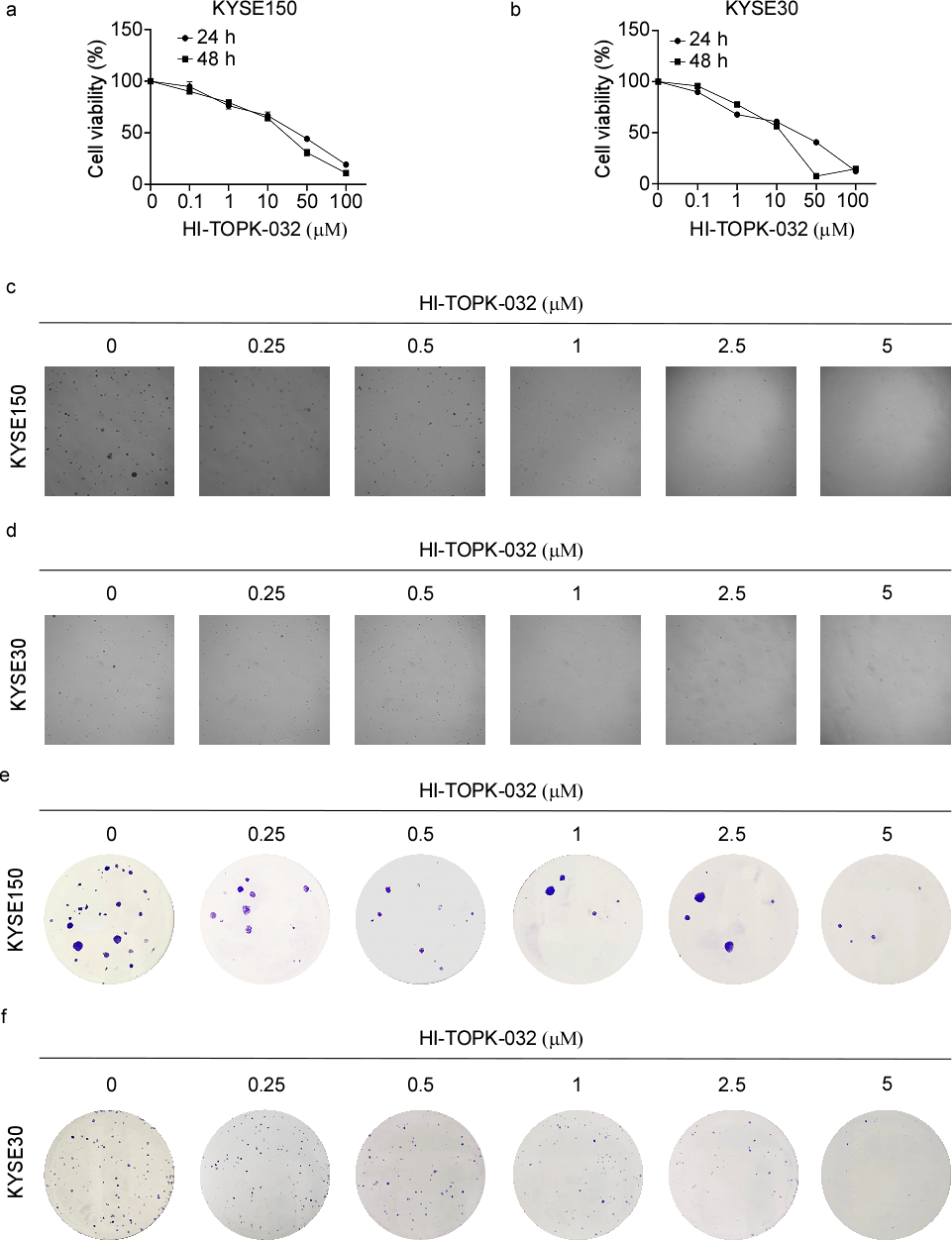


Supplemental Figure 6. HI-TOPK-032 inhibits EC cell proliferation *in vitro*. a b. Cytotoxicity of HI-TOPK-032 on KYSE150 (a) and KYSE30 (b) cells. Cells were treated with HI-TOPK-032 at various concentrations and then measured cell viability at 24 h and 48 h. c d. Representative images of anchorage-independent growth in HI-TOPK-032 treated KYSE150 (c) and KYSE30 (d) cells. KYSE150 and KYSE30 cells were treated with various concentrations of HI-TOPK-032 (0, 0.25, 0.5, 1, 2.5, 5 μM). e f. Representative images of anchorage-dependent growth in HI-TOPK-032 treated KYSE150 (e) and KYSE30 (f) cells. KYSE150 and KYSE30 cells were treated with various concentrations of HI-TOPK-032 (0, 0.25, 0.5, 1, 2.5, 5 μM). All experiments were biological replicates and were repeated at least three times.
